# Association between follow-up endoscopic inflammation and subsequent esophageal stricture formation after caustic ingestion

**DOI:** 10.1097/MD.0000000000050628

**Published:** 2026-09-11

**Authors:** Yoo Kyoung Lim, Heejoon Jang, Ji Won Kim

**Affiliations:** a Department of Internal Medicine, Seoul National University College of Medicine, Seoul Metropolitan Government Seoul National University Boramae Medical Center, Seoul, Republic of Korea.

**Keywords:** caustics, dilatation, esophageal stenosis, esophagitis, esophagoscopy

## Abstract

The prognostic accuracy of initial endoscopic findings using the Zargar classification for long-term outcomes remains imperfect. We evaluated whether mucosal inflammation on follow-up endoscopy provides additional prognostic information regarding the risk of esophageal stricture requiring therapeutic intervention. We included 20 patients with corrosive esophagitis who underwent initial endoscopy within 48 hours of caustic ingestion and follow-up endoscopy at Seoul Metropolitan Government Seoul National University Boramae Medical Center between January 1, 2010, and August 30, 2025. The outcome was esophageal stricture requiring balloon dilation or stent insertion. Patients were categorized by the presence or absence of esophageal inflammation on follow-up endoscopy, defined by persistent caustic-related mucosal inflammatory or injury findings, including hyperemia, edema, erosions or ulcerations, friability or bleeding, exudates, clots, or necrotic changes. Firth penalized Cox regression was used to identify predictors of endoscopic intervention. Follow-up endoscopy was performed at a median of 13 (interquartile range, 7–29) days after the initial examination. Seven patients (35%) demonstrated inflammatory findings on follow-up endoscopy, while 13 patients (65%) showed no inflammation. All 4 patients requiring endoscopic intervention had sustained inflammation on follow-up endoscopy, whereas none without inflammation required therapeutic intervention. In multivariable analysis, inflammation on follow-up endoscopy was associated with an increased risk of requiring endoscopic intervention, although the estimate was imprecise, with a wide confidence interval (adjusted hazard ratio, 17.14; 95% confidence interval, 1.67–2308.65; *P *= .014). Initial Zargar classification grades were not significantly associated with the outcome. Sustained mucosal inflammation on follow-up endoscopy may provide additional prognostic information regarding the risk of esophageal stricture requiring intervention in corrosive esophagitis. These findings warrant validation in larger prospective cohorts.

## 1. Introduction

Corrosive esophagitis is a severe form of upper gastrointestinal injury resulting from the ingestion of strong acids or alkalis. These injuries may lead to acute life-threatening complications, including perforation and mediastinitis, as well as chronic sequelae such as esophageal strictures. Etiology varies by age: pediatric cases are predominantly accidental, whereas adult cases are more commonly associated with intentional self-harm. Although the incidence has decreased in developed countries due to stricter regulations and increased public awareness, corrosive esophagitis remains a significant public health issue in developing regions where caustic substance management is inadequate.^[1–3]^

Clinical assessment and management of corrosive esophagitis remain challenging due to the unpredictable nature of tissue injury progression. Although the type of corrosive agent – acids causing coagulative necrosis and alkalis causing liquefactive necrosis – has traditionally guided clinical expectations, emerging evidence indicates that chemical properties alone do not reliably predict injury severity or clinical outcomes.^[1,4,5]^ Instead, multiple interacting factors, including patient age, agent concentration, ingested volume, duration of mucosal contact, and time to intervention, influence the clinical course, highlighting the need for dynamic and individualized assessment strategies.^[6,7]^

Endoscopic evaluation within 24–48 hours post-ingestion is currently considered the gold standard for assessing mucosal damage. The Zargar classification system, which stratifies injury severity from grade 0 (normal mucosa) to grade 4 (perforation), remains the most widely accepted and clinically utilized endoscopic grading system for caustic injury and is commonly used to guide management and estimate clinical outcomes.^[8]^ However, its correlation with the histopathological depth of injury is imperfect, particularly in intermediate-grade lesions, which may limit its prognostic accuracy for long-term outcomes.^[9]^ Computed tomography-based grading systems have also emerged as complementary or alternative approaches for prognosis, with some studies suggesting superior and others comparable predictive performance compared with Zargar-based endoscopic grading.^[10,11]^

Because mucosal injury continues to evolve after the initial insult, a single early assessment may not fully capture subsequent disease progression. Follow-up endoscopy may provide complementary prognostic information, but standardized criteria for follow-up assessment are lacking. We therefore evaluated the association between persistent mucosal inflammation on follow-up endoscopy and esophageal stricture requiring therapeutic intervention, together with initial injury severity and relevant clinical parameters.

## 2. Materials and methods

### 2.1. Study population

This study included patients who presented to the emergency department of Seoul Metropolitan Government–Seoul National University Boramae Medical Center following ingestion of a corrosive substance between January 1, 2010, and August 30, 2025. Eligible patients were required to have undergone endoscopic examination within 48 hours of ingestion and to have a confirmed diagnosis of corrosive esophagitis. Patients were excluded if the ingested substance could not be identified or if follow-up endoscopic evaluation was not performed after the initial diagnosis.

### 2.2. Study design and outcome

Patients were categorized according to the presence or absence of esophageal inflammation on follow-up endoscopy. Esophageal inflammation on follow-up endoscopy was operationally defined as the presence of 1 or more persistent caustic-related mucosal inflammatory or injury findings, including hyperemia or hemorrhagic changes, edema, erosions or ulcerations, mucosal friability or spontaneous bleeding, exudates, thick mucus or blood clots, and necrotic mucosal changes. Follow-up endoscopic images were reviewed by 2 experienced endoscopists, with the final classification determined by consensus. Reviewers were not provided with subsequent outcome data at the time of image review. The timing of follow-up endoscopy was based on clinical judgment rather than a predefined protocol, guided by the patient’s symptoms and clinical course. The primary outcome was the development of an esophageal stricture requiring therapeutic intervention, including balloon dilation or stent placement.

Demographic and clinical data were systematically extracted from electronic medical records. Variables collected included age, sex, type of corrosive agent, severity of esophageal damage based on the Zargar classification, presence of laryngeal edema, pH and estimated volume of the ingested substance, circumstances of ingestion (intentional vs accidental), time interval from ingestion to initial endoscopy, presence of vomiting, timing of follow-up examination, and baseline laboratory parameters (white blood cell [WBC] count, hemoglobin, aspartate aminotransferase, alanine aminotransferase, total bilirubin, and creatinine). Corrosive agents were classified according to pH as follows: strong acids (pH < 3.0), weak acids (pH 3.0–6.9), weak alkalis (pH 7.1–11.0), and strong alkalis (pH > 11.0). Extreme pH values (<3.0 and >11.0) were considered to confer the highest risk for tissue damage.^[12]^

This study was conducted in accordance with the 2013 revision of the Declaration of Helsinki and the International Council for Harmonisation Good Clinical Practice guidelines and was approved by the Institutional Review Board of Seoul Metropolitan Government Seoul National University Boramae Medical Center (No. 10-2025-42). Data were accessed for research purposes between September 6, 2025, and September 30, 2025.

### 2.3. Statistical analysis

Normality of continuous variables was tested using the Shapiro–Wilk test. Normally distributed data are presented as mean ± standard deviation, and non-normally distributed data as medians with interquartile ranges (IQR). Categorical variables are expressed as counts and percentages. Group comparisons were made using appropriate statistical tests based on variable type and distribution. To identify clinical predictors of endoscopic intervention, univariable and multivariable survival analyses were conducted using Firth penalized Cox proportional hazards regression, which is appropriate for small sample sizes and rare events.^[13,14]^ Time-to-event was defined as the interval from initial endoscopy to endoscopic intervention. Kaplan–Meier curves were compared using the log-rank test. Univariable analyses were first conducted for each clinical variable. Hazard ratios (HRs) with corresponding 95% confidence intervals (CIs) were estimated using profile-likelihood methods. Variables with a *P* value <.05 in univariable analyses were included in the multivariable model. For categorical variables, the most clinically relevant category served as the reference. Continuous variables were analyzed per unit increase; WBC count was rescaled and analyzed per 1000 cells/µL increase to facilitate clinical interpretation. All statistical analyses were performed using R software, version 4.5 (R Foundation for Statistical Computing).

## 3. Results

**Figure 1.**
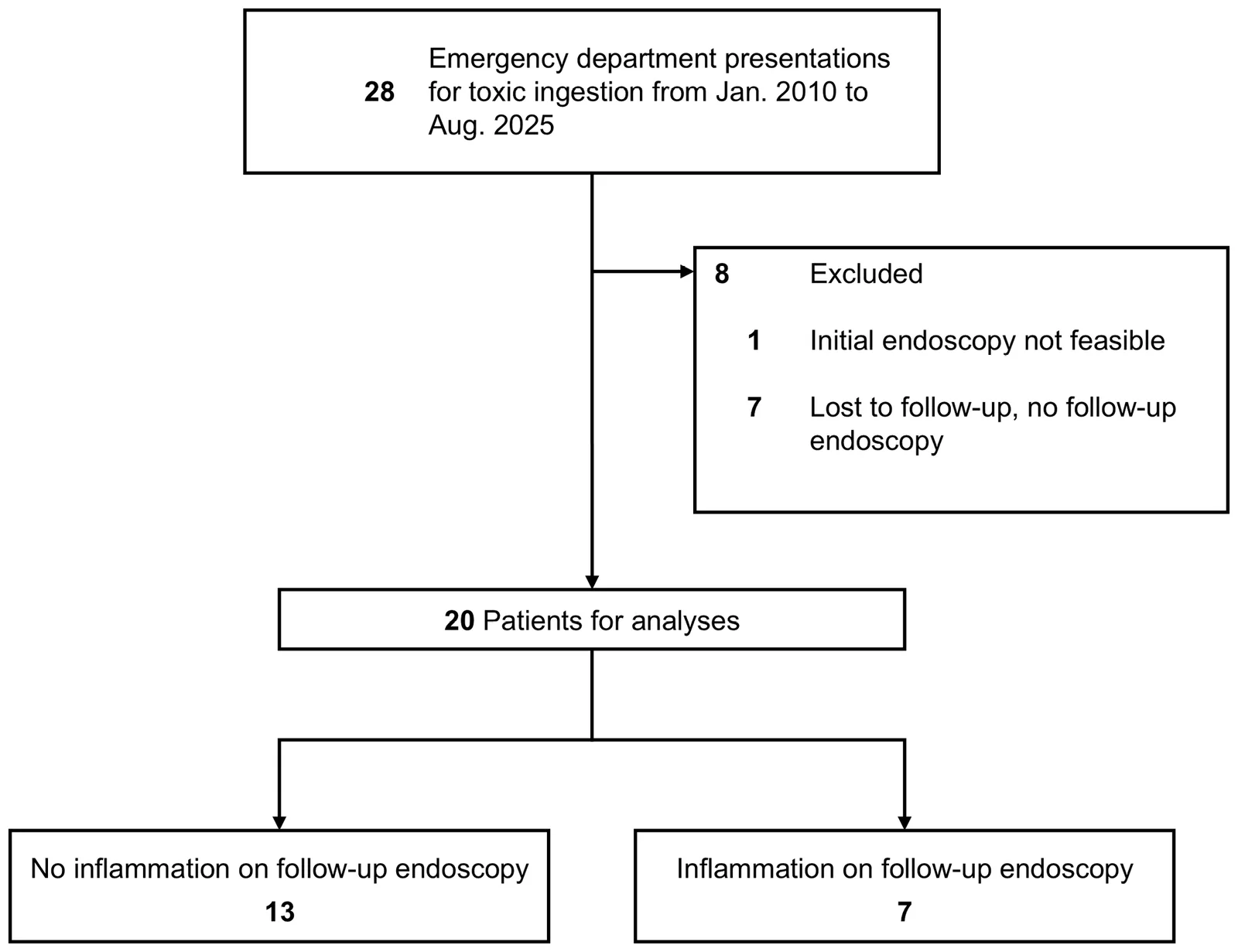
Study population.

Among 28 patients presenting to the emergency department following caustic ingestion, 1 patient who did not undergo initial emergency endoscopy and 7 who did not undergo follow-up endoscopy were excluded. Consequently, 20 patients were included in the final analysis (Fig. 1). Baseline characteristics of the 7 patients excluded because they did not undergo follow-up endoscopy are presented in Table S1, Supplemental Digital Content 1. The median time from ingestion to initial emergency endoscopy was 6.0 hours (IQR, 3.8–8.1), and the median interval between initial and follow-up endoscopy was 13 days (IQR, 7–29). The median duration of clinical follow-up from the initial endoscopy was 1308 days (IQR, 77–2254), providing substantial longitudinal data for outcome assessment.

**Figure 2.**
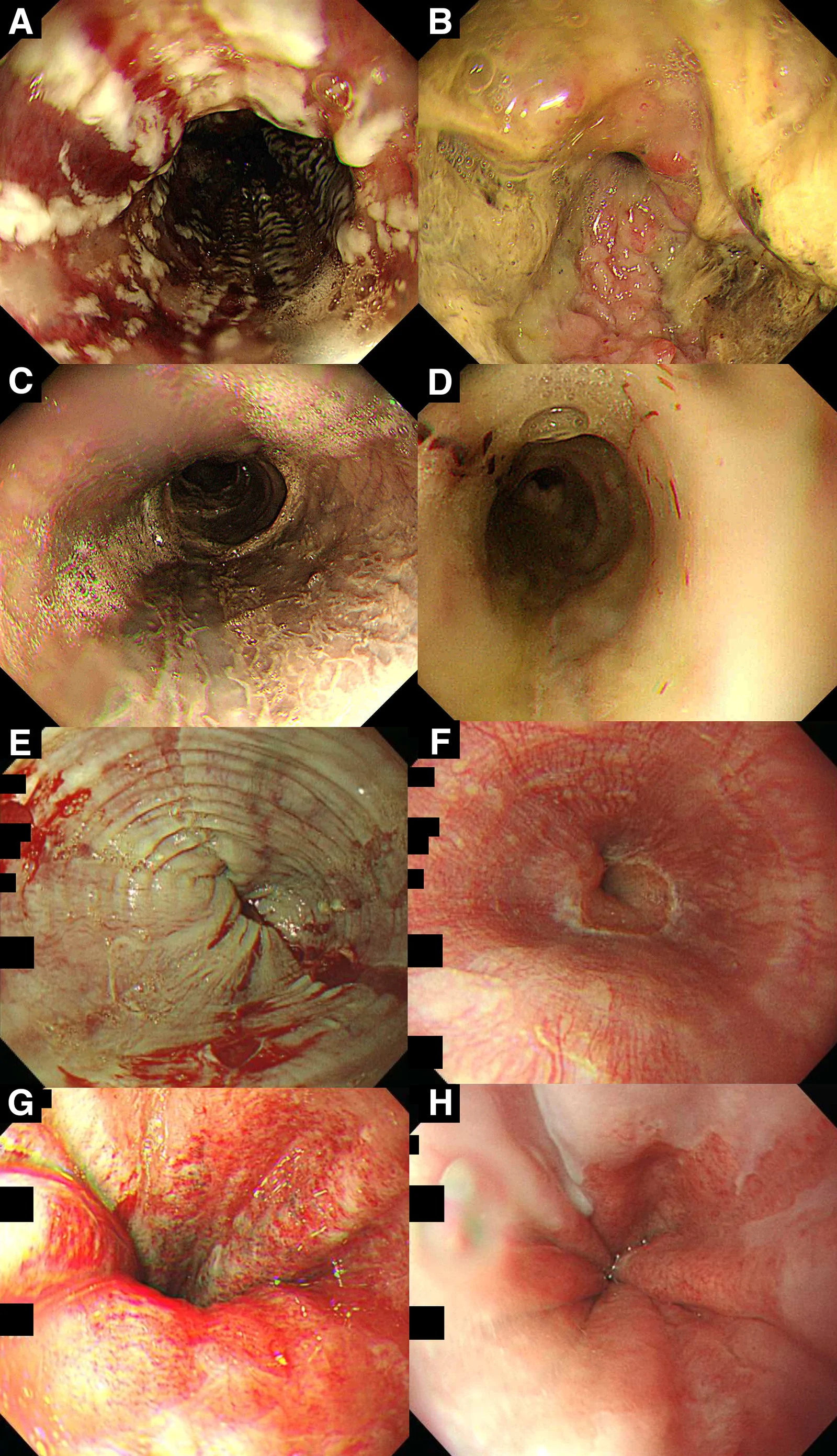
Representative serial endoscopic findings according to the presence or absence of persistent mucosal inflammation on follow-up endoscopy. (A–D) Patients with persistent mucosal inflammation on follow-up endoscopy after strong acid ingestion (A and B) and strong alkali ingestion (C and D). (A) Initial esophagogastroduodenoscopy (EGD) showing caustic esophageal injury with focal mucosal necrosis and areas of circumferential involvement. (B) Follow-up EGD showing persistent mucosal inflammation with edema, patchy hyperemia, residual ulceration, and extensive adherent yellow-white exudate. (C) Initial EGD showing diffuse caustic esophageal injury with dark necrotic mucosa and mucosal sloughing. (D) Follow-up EGD showing persistent mucosal inflammation characterized by residual erythema and longitudinal erosive-ulcerative changes. (E–H) Patients without persistent mucosal inflammation on follow-up endoscopy after strong acid ingestion (E and F) and strong alkali ingestion (G and H). (E) Initial EGD showing diffuse caustic esophageal injury with pale-whitish mucosal discoloration, areas of circumferential involvement, and hemorrhagic changes. (F) Follow-up EGD showing substantial mucosal healing without definite persistent caustic-related inflammatory changes. (G) Initial EGD showing diffuse caustic esophageal injury with marked erythema, mucosal edema, and erosive-exudative changes. (H) Follow-up EGD showing substantial mucosal healing without definite persistent caustic-related inflammatory changes.

Patients were stratified according to the presence of mucosal inflammation on follow-up endoscopy: 13 patients (65%) had no inflammation, whereas 7 patients (35%) exhibited signs of inflammation. Representative follow-up endoscopic findings with and without persistent mucosal inflammation are shown in Figure 2. As Table 1 shows, baseline characteristics did not differ significantly between the 2 groups. Sex distribution was comparable (male: 43% vs 46%, *P* > .99), as was mean age (60 ± 21 vs 47 ± 22 years, *P* = .24). Initial Zargar grade (*P* = .36) and the presence of laryngeal edema (43% vs 15%, *P* = .29) were also comparable. The median time from ingestion to initial endoscopy did not differ significantly between groups (5.0 vs 7.0 hours, *P* = .75). Although not statistically significant, some clinically relevant trends were observed: alkali ingestion was less common in the inflammation group (29% vs 77%, *P* = .06), whereas strong acid ingestion was more frequent (71% vs 15%, *P* = .08). Other clinical variables, including vomiting, intentional ingestion, and time to follow-up endoscopy, were comparable between groups. Laboratory parameters at presentation, including WBC count, hemoglobin level, liver enzymes (aspartate aminotransferase, alanine aminotransferase), total bilirubin, and serum creatinine, also demonstrated no significant between-group differences.

**Table 1:** Patient characteristics by presence of inflammation on follow-up endoscopy.

Characteristic	Overall	Inflammation on follow-up endoscopy		*P*
		Absent	Present	
	(n = 20)	(n = 13)	(n = 7)	
Male	9 (45%)	6 (46%)	3 (43%)	>.99
Age	51 ± 22	47 ± 22	60 ± 21	.24
Zargar classification				.36
1	4 (20%)	3 (23%)	1 (14%)	
2A	11 (55%)	8 (62%)	3 (43%)	
3A	3 (15%)	2 (15%)	1 (14%)	
3B	2 (10%)	0 (0%)	2 (29%)	
Laryngeal edema	5 (25%)	2 (15%)	3 (43%)	.29
Time to endoscopy, h	6.0 (3.8–8.1)	7.0 (3.0–9.0)	5.0 (4.1–6.9)	.75
Alkali ingestion	12 (60%)	10 (77%)	2 (29%)	.06
Acidity*				.08
Strong acid	7 (35%)	2 (15%)	5 (71%)	
Weak acid	1 (5.0%)	1 (7.7%)	0 (0%)	
Weak alkali	3 (15%)	3 (23%)	0 (0%)	
Strong alkali	9 (45%)	7 (54%)	2 (29%)	
Amount ingested, mL	100 (31–195)	165 (44–250)	65 (31–95)	.22
Vomiting	12 (60%)	7 (54%)	5 (71%)	.64
Intentional ingestion	15 (75%)	9 (69%)	6 (86%)	.61
Time to follow-up endoscopy, d	13 (7–29)	20 (7–33)	8 (7–13)	.12
WBC count, ×10^3^/μL	11.6 (8.4–18.3)	11.3 (8.3–14.6)	17.2 (10.8–23.9)	.24
Hemoglobin, g/dL	14.17 ± 1.38	14.38 ± 1.13	13.76 ± 1.77	.58
Aspartate aminotransferase, IU/L	29 (21–34)	29 (21–35)	30 (20–33)	.75
Alanine aminotransferase, IU/L	14 (11–20)	15 (11–23)	13 (11–18)	.72
Total bilirubin, mg/dL	1.05 (0.63–1.40)	1.10 (0.70–1.40)	1.00 (0.70–1.15)	.58
Creatinine, mg/dL	0.77 ± 0.14	0.77 ± 0.14	0.76 ± 0.15	>.99

Data are presented as mean ± standard deviation, median (interquartile range), or number (%), as appropriate.

WBC = white blood cell.

* Acidity was categorized according to pH as strong acid (pH < 3.0), weak acid (pH 3.0–6.9), weak alkali (pH 7.1–11.0), and strong alkali (pH > 11.0).

### 3.1. Esophageal stricture according to follow-up endoscopic inflammation

**Figure 3.**
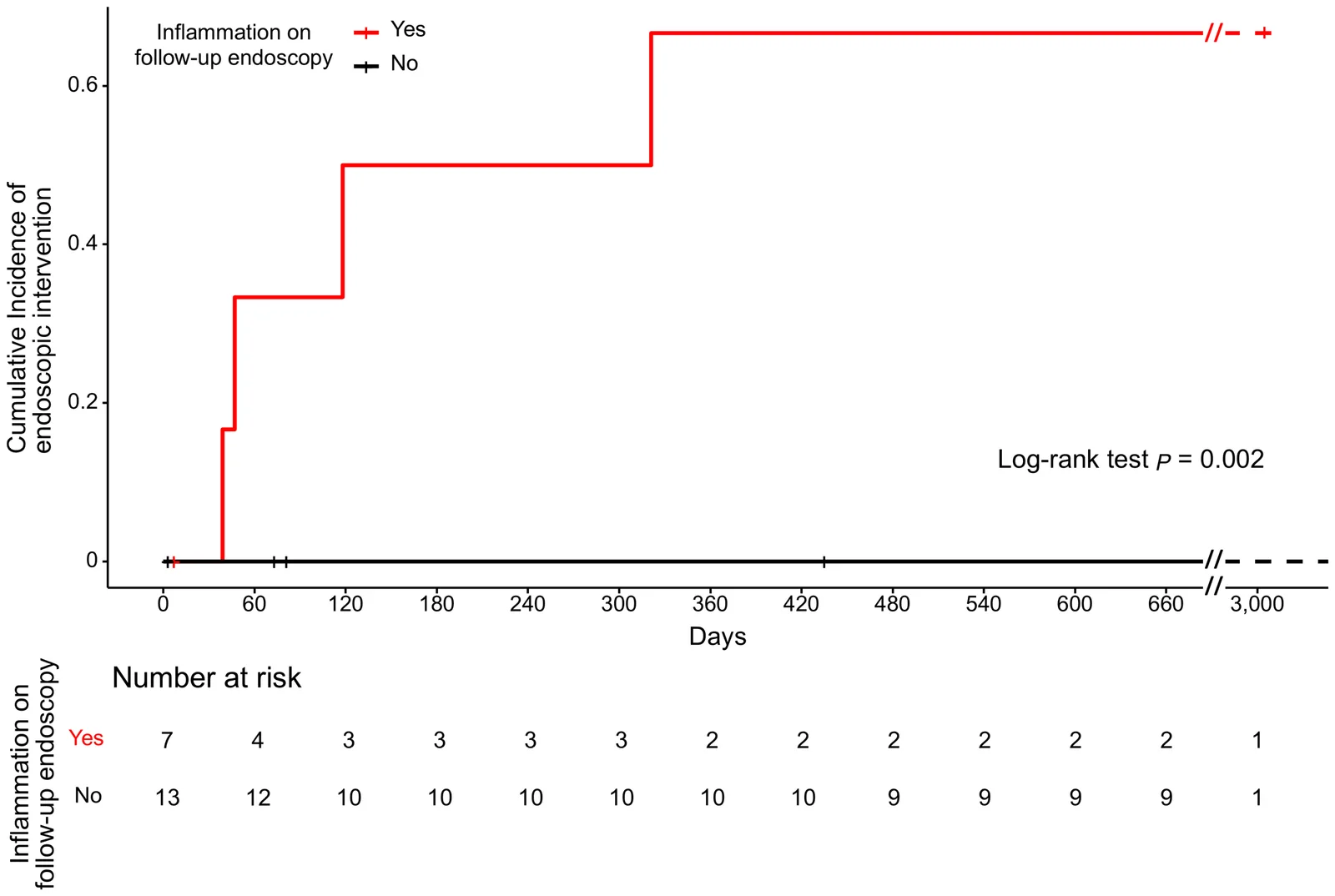
Cumulative incidence of endoscopic intervention according to esophageal inflammatory findings on follow-up endoscopy.

Endoscopic intervention was required in 4 patients, all of whom demonstrated mucosal inflammation on follow-up endoscopy. Initial endoscopic findings in these patients revealed Zargar classification grade 2A in 1 patient, 3A in 1 patient, and 3B in 2 patients. Among them, 3 patients underwent balloon dilation, and 1 underwent esophageal stent placement. Follow-up endoscopy was performed at a median of 13 days (IQR, 7–29) after the initial examination, with no significant difference between patients with inflammation (8 days [IQR, 7–13]) and those without inflammation (20 days [IQR, 7–33]) (*P* = .12). The median time to stricture detection was 32 days (IQR, 22–41) after the initial examination, and the median interval from initial endoscopy to endoscopic intervention was 83 days (IQR, 45–169). Kaplan–Meier survival curves (Fig. 3) illustrate the cumulative incidence of endoscopic intervention over time. Patients with mucosal inflammation on follow-up endoscopy had a significantly higher cumulative incidence of subsequent therapeutic endoscopic intervention (log-rank *P* = .002).

### 3.2. Survival analysis using Firth penalized Cox regression

Univariable and multivariable survival analyses using Firth penalized Cox regression are presented in Table 2. In the univariable analysis using Firth penalized Cox regression, mucosal inflammation on follow-up endoscopy was significantly associated with an increased risk of endoscopic intervention (HR, 23.07; 95% CI, 2.44–612.66; *P* = .004). WBC count was also associated with a higher risk of intervention when analyzed per 1000 cells/µL increase (HR, 1.11; 95% CI, 1.00–1.24; *P* = .04). Other clinical factors, including age, sex, Zargar grade, presence of laryngeal edema, type and amount of corrosive substance ingested, vomiting, and timing of endoscopy, were not significantly associated with the outcome in univariable analysis (all *P* > .05). A multivariable Firth Cox regression model incorporating the 2 variables with *P* < .05 in univariable analysis was subsequently constructed. In the adjusted model, mucosal inflammation on follow-up endoscopy remained associated with endoscopic intervention (HR, 17.14; 95% CI, 1.67–2308.65; *P* = .014), whereas WBC count was no longer statistically significant (HR, 1.07; 95% CI, 0.97–1.21; *P* = .19).

**Table 2:** Factors associated with endoscopic intervention by Firth penalized Cox regression.

Variable	Level	Univariable HR (95% CI)	Multivariable HR (95% CI)
Inflammation on follow-up endoscopy	No	Reference	
	Yes	23.07 (2.44–612.66)	17.14 (1.67–2308.65)
Sex	Female	Reference	
	Male	0.45 (0.05–3.81)	
Age		1.00 (0.96–1.04)	
Zargar classification	1	Reference	
	2A	1.55 (0.03–78.83)	
	3A	7.62 (0.15–398.74)	
	3B	12.21 (0.29–514.04)	
Laryngeal edema	No	Reference	
	Yes	2.04 (0.24–17.52)	
Time to endoscopy, h		1.04 (0.91–1.19)	
Alkali ingestion	No	Reference	
	Yes	0.59 (0.08–4.19)	
Acidity*	Strong acid	Reference	
	Weak acid	0.92 (0.02–38.40)	
	Weak alkali	0.31 (0.01–12.80)	
	Strong alkali	0.71 (0.08–6.06)	
Amount ingested, mL		1.00 (0.99–1.01)	
Vomiting	No	Reference	
	Yes	2.07 (0.24–17.56)	
Intentional ingestion	No	Reference	
	Yes	3.70 (0.14–97.22)	
Time to follow-up endoscopy, d		0.94 (0.84–1.05)	
WBC count, per × 10^3^/μL		1.11 (1.00–1.24)	1.07 (0.97–1.21)
Hemoglobin, g/dL		0.92 (0.46–1.84)	
Aspartate aminotransferase, IU/L		1.00 (0.95–1.04)	
Alanine aminotransferase, IU/L		0.98 (0.88–1.10)	
Total bilirubin, mg/dL		0.99 (0.56–1.75)	
Creatinine, mg/dL		0.17 (0.00–280.68)	

CI = confidence interval, HR = hazard ratio, WBC = white blood cell.

* Acidity was categorized according to pH as strong acid (pH < 3.0), weak acid (pH 3.0–6.9), weak alkali (pH 7.1–11.0), and strong alkali (pH > 11.0).

## 4. Discussion

This study indicates that mucosal inflammation observed on follow-up endoscopy may serve as a prognostic marker for esophageal stricture requiring intervention in patients with corrosive esophagitis. All patients who ultimately required endoscopic treatment exhibited mucosal inflammation on follow-up endoscopy performed at a median of 8 days (IQR, 7–13) after the initial examination, whereas none of the patients without inflammation on follow-up endoscopy required therapeutic intervention. In multivariable analysis, the presence of inflammation on follow-up endoscopy was associated with an increased risk of requiring endoscopic intervention (HR, 17.14; 95% CI, 1.67–2308.65; *P* = .014). Although the wide confidence interval reflects limited precision, the observed association in our cohort suggests that persistent mucosal inflammation on follow-up endoscopy may provide clinically relevant prognostic information beyond initial injury severity.

The traditional management of corrosive esophagitis has relied primarily on initial endoscopic findings using the Zargar classification system to predict clinical outcomes.^[8,15]^ While this system has served as the cornerstone of risk stratification since its introduction in 1989, efforts to improve prognostic accuracy have focused on incorporating additional baseline clinical parameters, including laboratory values, clinical presentations, and imaging findings.^[16–19]^ However, the prognostic value of follow-up endoscopic findings has received little attention in the literature. This represents an important gap, as corrosive esophagitis involves evolving mucosal pathology that may not be fully characterized by acute-phase evaluation alone.^[20]^ Our findings further suggest that persistent mucosal inflammation on follow-up endoscopy may provide complementary prognostic information for identifying patients at risk of subsequent stricture requiring intervention.

Although systematic follow-up endoscopy has not been extensively studied in corrosive esophagitis, its potential utility is supported by evidence from other inflammatory esophageal conditions. Recent studies in eosinophilic esophagitis have demonstrated that close endoscopic monitoring is associated with reduced stricture formation and earlier detection of complications.^[21]^ A similar approach may be applicable to corrosive esophagitis, given shared inflammatory mechanisms and the potential for stricture development in both conditions. While concerns regarding iatrogenic perforation may discourage the use of follow-up endoscopy during the subacute phase, multiple studies have demonstrated that repeat endoscopy can be safely performed within 3 weeks of ingestion when conducted by experienced endoscopists.^[5,22,23]^ Consistent with these findings, no procedure-related complications occurred in our cohort, further supporting the safety of follow-up endoscopy in corrosive esophagitis.

The timing of follow-up endoscopy may be clinically relevant because mucosal healing evolves dynamically during the subacute phase after caustic ingestion. Previous studies indicate that mucosal sloughing may persist for up to 7 days after caustic ingestion, whereas acute inflammation typically resolves and reepithelialization begins around 10 to 15 days post-injury.^[20]^ In our study, mucosal inflammation was observed on follow-up endoscopy performed at a median of 8 days after the initial examination and was significantly associated with the subsequent need for endoscopic intervention. Notably, 1 patient initially classified as Zargar grade 2A exhibited inflammatory changes on follow-up endoscopy and subsequently required therapeutic intervention, illustrating how the healing process may reveal prognostically important changes not apparent during initial assessment. These findings support the potential clinical utility of early follow-up endoscopy for risk stratification in corrosive esophagitis. Further prospective validation is needed before this approach can be incorporated into routine clinical practice.

Some limitations should be acknowledged when interpreting these results. First, the retrospective design and small sample size (n = 20), with only 4 outcome events, limit statistical power and the precision of the effect estimates. The small cohort reflects, in part, the rarity of corrosive esophagitis requiring both initial and follow-up endoscopic evaluation, despite a study period of >15 years at a large university-affiliated hospital. To mitigate these challenges, we used Firth penalized Cox regression to reduce small-sample bias associated with sparse events. Second, selection bias may have been introduced by excluding patients who did not undergo follow-up endoscopy. Although the overall distribution of Zargar grades did not differ significantly between included and excluded patients, the excluded patients showed a numerical tendency toward less severe initial endoscopic injury and underwent initial endoscopy later after ingestion. These differences suggest that the study cohort may not fully represent the broader population of patients with corrosive esophagitis. Future prospective studies using standardized follow-up protocols are needed to minimize this potential bias. Third, assessment of inflammation on follow-up endoscopy was inherently subjective. Images were reviewed by consensus between 2 experienced endoscopists who were not provided with subsequent outcome data; however, the consensus-based approach precluded formal assessment of interobserver agreement. Future studies using standardized criteria and independent blinded review are needed to improve reproducibility. Fourth, the timing of follow-up endoscopy was not standardized but was determined by clinical judgment, resulting in substantial variability (median 13 days; IQR, 7–29 days). Because mucosal healing after caustic injury is dynamic, differences in follow-up timing may have influenced the classification of persistent inflammation. Standardized timing of follow-up endoscopy in future prospective studies will be important to clarify the prognostic significance of these findings. Finally, the single-center design may limit external validity due to potential changes in clinical practice and patient demographics over time. Multicenter studies involving diverse populations are required to validate these findings across different demographic groups and healthcare settings.

## 5. Conclusion

Sustained esophageal mucosal inflammation on follow-up endoscopy may be associated with an increased risk of esophageal stricture requiring intervention in corrosive esophagitis. Follow-up endoscopic assessment may provide complementary prognostic information beyond the initial evaluation. Further prospective studies are needed to validate these findings and standardize follow-up endoscopic assessment.

## Author contributions

**Conceptualization:** Heejoon Jang, Ji Won Kim.

**Data curation:** Yoo Kyoung Lim, Heejoon Jang.

**Formal analysis:** Yoo Kyoung Lim, Heejoon Jang.

**Investigation:** Yoo Kyoung Lim.

**Methodology:** Heejoon Jang.

**Project administration:** Ji Won Kim.

**Resources:** Ji Won Kim.

**Software:** Heejoon Jang.

**Supervision:** Heejoon Jang, Ji Won Kim.

**Visualization:** Yoo Kyoung Lim.

**Writing – original draft:** Yoo Kyoung Lim.

**Writing – review & editing:** Heejoon Jang, Ji Won Kim.


