# Supplementary material for: Association between follow-up endoscopic inflammation and subsequent esophageal stricture formation after caustic ingestion
Source: Medicine (Baltimore). 2026 Sep 11;105(37):e50628. doi: 10.1097/MD.0000000000050628 (PMC13574382; doi:10.1097/MD.0000000000050628)
Supplement: Supplementary file 1 [file medi-105-e50628-s001.docx]

Supplementary Table 1. Baseline characteristics according to performance of follow-up endoscopy

| Characteristic | Follow-up EGD performed (n = 20) | Follow-up EGD not performed (n = 7) | *P* |
| --- | --- | --- | --- |
| Male | 9 (45%) | 5 (71%) | 0.39 |
| Age | 51.5 ± 22.0 | 47.9 ± 24.5 | 0.74 |
| Zargar classification |  |  | 0.14 |
| 1 | 4 (20%) | 5 (71%) |  |
| 2A | 11 (55%) | 2 (29%) |  |
| 3A | 3 (15%) | 0 (0%) |  |
| 3B | 2 (10%) | 0 (0%) |  |
| Laryngeal edema | 5 (25%) | 0 (0%) | 0.28 |
| Time to initial endoscopy, hour | 6.0 (3.8–8.1) | 26.0 (21.5–39.5) | <0.01 |
| Alkali ingestion | 12 (60%) | 6 (86%) | 0.36 |
| Acidity* |  |  | 0.23 |
| Strong acid | 7 (35%) | 0 (0%) |  |
| Weak acid | 1 (5%) | 1 (14%) |  |
| Weak alkali | 3 (15%) | 1 (14%) |  |
| Strong alkali | 9 (45%) | 5 (71%) |  |
| Amount ingested, mL | 100 (31–195) | 100 (30–138) | >0.99 |
| Vomiting | 12 (60%) | 1 (14%) | 0.08 |
| Intentional ingestion | 15 (75%) | 2 (29%) | 0.07 |
| WBC count, ×10³/μL | 11.6 (8.4–18.3) | 8.4 (7.2–9.8) | 0.13 |
| Hemoglobin, g/dL | 14.2 ± 1.4 | 13.5 ± 1.0 | 0.25 |
| Aspartate aminotransferase, IU/L | 29 (21–34) | 24 (20–30) | 0.68 |
| Alanine aminotransferase, IU/L | 14 (11–20) | 20 (11–26) | 0.5 |
| Total bilirubin, mg/dL | 1.05 (0.63–1.40) | 0.80 (0.80–1.10) | 0.97 |
| Creatinine, mg/dL | 0.77 ± 0.14 | 0.80 ± 0.07 | 0.51 |

Data are presented as mean ± standard deviation, median (interquartile range), or number (%), as appropriate.

*Acidity was categorized according to pH as strong acid (pH < 3.0), weak acid (pH 3.0–6.9), weak alkali (pH 7.1–11.0), and strong alkali (pH > 11.0).

Abbreviations: EGD, esophagogastroduodenoscopy; WBC, white blood cell.
